# Targeting Survivin: Now I Become Death, the Destroyer of Cells

**DOI:** 10.3390/ijms262311417

**Published:** 2025-11-26

**Authors:** Mia Fanuzzi, Shuhua Zheng, Craig M. Horbinski, Maryam A. Shaaban, Harrshavasan Congivaram, Ruochen Du, Shashwat Tripathi, Lisa Hurley, Priya Kumthekar, Atique Ahmed, Daniel J. Brat, Maciej S. Lesniak, Amy B. Heimberger

**Affiliations:** 1 Department of Neurological Surgery, Feinberg School of Medicine, Northwestern University, Chicago, IL 60611, USA; mia.fanuzzi@northwestern.edu (M.F.); horbinski.craig@mayo.edu (C.M.H.); maryams2@uic.edu (M.A.S.); harrshavasan.congivaram@northwestern.edu (H.C.); ruochen.du@northwestern.edu (R.D.); shashwat.tripathi@northwestern.edu (S.T.); l-hurley@northwestern.edu (L.H.); atique.ahmed@northwestern.edu (A.A.); maciej.lesniak@northwestern.edu (M.S.L.); 2 Malnati Brain Tumor Institute of the Robert H. Lurie Comprehensive Cancer Center, Feinberg School of Medicine, Northwestern University, Chicago, IL 60611, USA; shuhua.zheng@northwestern.edu (S.Z.); priya.kumthekar@nm.org (P.K.); daniel.brat@northwestern.edu (D.J.B.); 3 Department of Radiation Oncology, Robert H. Lurie Comprehensive Cancer Center of Northwestern University, Chicago, IL 60611, USA; 4 Department of Neurology, Feinberg School of Medicine, Northwestern University, Chicago, IL 60611, USA; 5 Department of Pathology, Feinberg School of Medicine, Northwestern University, Chicago, IL 60611, USA

**Keywords:** apoptosis, survivin, glioblastoma, breast cancer

## Abstract

Survivin (*BIRC*5) plays a key role in inhibiting apoptosis and is highly expressed in many cancers, including gliomas and breast cancer, where it contributes to tumor progression, therapeutic resistance and poor patient outcomes. With a dual function in promoting cell proliferation and survival, coupled with its potential immunogenicity, survivin is a compelling therapeutic target for cancer; yet, it has no FDA-approved agents to date. Here, we review key findings from preclinical models that emphasize how survivin contributes to chemoresistance and radioresistance; summarize the clinical landscape of survivin-targeted strategies, highlighting both the successes and limitations of these approaches; and outline next steps to optimize survivin-targeted therapies, including the need to integrate biomarker-focused patient selection and the potential for combination therapies. These insights establish survivin as a key driver of cancer progression and a promising target for future therapeutic development.

## 1. The Role of Survivin in Mediating Cell Cycle and Death

**Figure 1:**
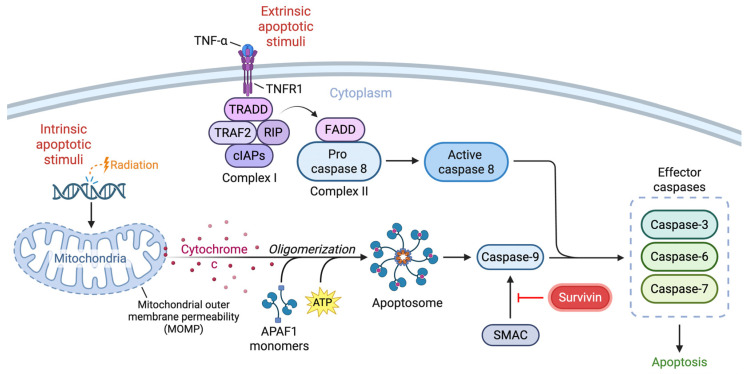
Schematic illustrating the intrinsic and extrinsic pathways of apoptosis and the regulation of these events by survivin. The intrinsic pathway is triggered by stimuli like radiation, leading to mitochondrial outer membrane permeabilization (MOMP) and release of cytochrome c. Apoptotic protease activating factor 1 (APAF-1) monomers bind to cytochrome c with ATP co-factors and oligomerize, forming the apoptosome and activating caspase-9. In the extrinsic pathway, ligand binding of TNFR1 triggers the formation of complex I, which includes TRAF2, TRADD, RIP, and cIAPs. Complex II forms with TRADD, RIP, FADD and procaspase-8. Initiator caspases converge to activate effector caspases, leading to cell death. Survivin acts on the intrinsic pathway by disrupting the pro-apoptotic function of SMAC.

Survivin, encoded by *BIRC5*, is a multifunctional protein that regulates both apoptosis and the cell cycle. Cytosolic survivin acts as an inhibitor of apoptosis, whereas its nuclear form acts as a cell cycle regulator [1]. As a member of the inhibitor of apoptosis (IAP) protein family, survivin indirectly promotes cell survival in proliferating cells by blocking upstream apoptotic events, thereby reducing caspase activation. Apoptosis is initiated by intrinsic and extrinsic pathways that have convergent mechanisms. The intrinsic mitochondrial pathway is activated by internal cellular stress, such as DNA damage from ionizing radiation. In response to these stimuli, activated pro-apoptotic proteins, Bax and Bak, oligomerize and form pores in the mitochondrial outer membrane, causing release of cytochrome c and the formation of the apoptosome, a wheel-like structure that directs subsequent activation of caspase-9 [2]. In contrast, the extrinsic pathway is triggered by ligand binding to death receptors, such as tumor necrosis factor receptors (TNFR), including TNFR1. The interaction between tumor necrosis factor and TNFR1 triggers the formation of Complex I, consisting of tumor necrosis factor receptor-associated factor 2 (TRAF2), TNFR1-associated death domain protein (TRADD), receptor-interacting protein (RIP), and the cellular inhibitor of apoptosis proteins (cIAPs) [3]. TRADD and RIP are recruited by TNFR1 through homomeric death domain interactions, followed by the recruitment of TRAF2 and cIAPs by TRADD to form Complex I. Complex I formation mainly activates the NF-κB and MAPK pathways that regulate inflammation, immune responses and cell proliferation. Although a pro-apoptotic complex, the cIAPs present in this complex are responsible for the downstream activation of the MAPK pathway. Since Complex I does not directly trigger apoptosis, the formation of Complex II, which occurs when TRADD and RIP interact with Fas-associated death domain protein (FADD) and procaspase-8, is required. Caspase-8 is then activated, ultimately leading to the downstream convergence with the intrinsic pathway [3]. Both pathways activate effector caspases 3, 6, and 7, thereby triggering cell death. In the intrinsic pathway, survivin can bind to and inhibit the secondary mitochondria-derived activator of caspase (SMAC), a pro-apoptotic protein that normally neutralizes other IAPs. Upon binding to survivin, SMAC is released from the mitochondria but is unable to interact with IAPs or activate caspase-9 [1]. Survivin exerts its effects primarily through the intrinsic pathway to prevent apoptotic cell death (Figure 1). Beyond its role in apoptosis inhibition, survivin is critical for proper chromosome segregation and cytokinesis during mitosis, directly linking it to cell proliferation [4].

Cell cycle dysfunction is a hallmark of cancer and is a key driver of tumorigenesis, characterized by the disruption of checkpoint controls that normally ensure proper cell division. Mutations in genes that regulate critical cell cycle checkpoints, such as p53 and Rb, enable cells to bypass growth arrest signals and evade programmed cell death. Proteins like survivin mediate cell cycle dysfunction by promoting aberrant cell division. Tightly regulated through the cell cycle, survivin expression peaks during the G2/M phase and declines during the G1 phase, aligning with its function in mitotic regulation. During mitosis, survivin localizes to the mitotic spindle, where it interacts with tubulin to stabilize microtubules and ensure proper chromosome segregation [5]. Survivin is also a key component of the chromosomal passenger complex, which includes aurora-B kinase, the inner centromere protein, and borealin [6]. This complex controls critical mitotic events, including chromosome alignment and cytokinesis. As such, loss of survivin, either through biological selection or therapeutic targeting, may disrupt survivin-mediated functions and induce the sensitization of cells to apoptosis.

## 2. Role of Survivin in Cancer

Survivin overexpression is strongly associated with aggressive tumor growth, poor prognosis, and resistance to standard treatments, including chemotherapy and radiation. Many clinical studies have identified survivin as a robust prognostic indicator across cancer lineages, with elevated expression correlating with worse overall survival and reduced treatment efficacy [7]. Mechanistically, survivin drives tumorigenesis through its dual role of inhibiting apoptosis and regulating cell division. Its expression is regulated by several transcriptional and post-transcriptional mechanisms. Specifically, the activation of certain receptor tyrosine kinases, including EGFR, HER2, and IGF-1R, can upregulate survivin via the downstream PI3K/Akt [8], STAT3, and Wnt/β-catenin pathways [4]. Additionally, various miRNAs have been identified that modulate survivin expression by binding to the 3′-UTR of survivin mRNA, leading to altered protein or mRNA degradation. Moreover, post-translational modifications such as Thr34 phosphorylation promote survivin’s stability during metaphase, whereas its degradation is controlled by the ubiquitin–proteasome pathway through a cell-cycle-dependent manner [4]. As such, survivin has been widely used as a biomarker for cancer progression and a potential therapeutic target for decades.

**Figure 2:**
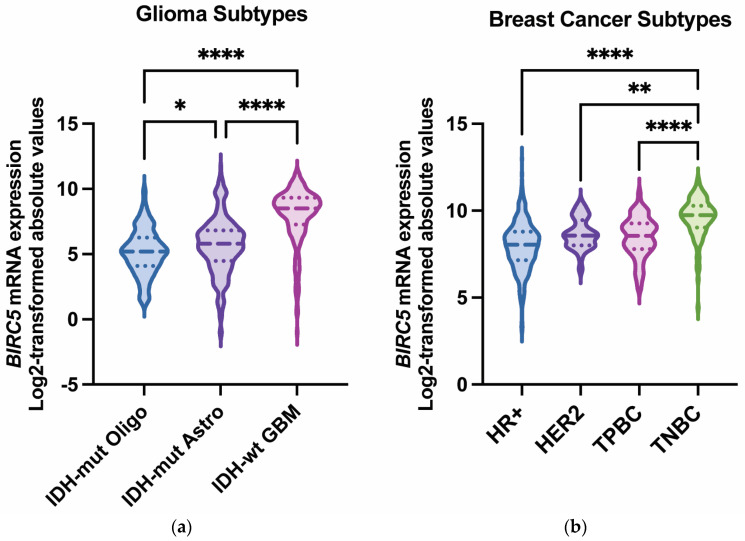
*BIRC5* (survivin) mRNA expression across glioma and breast cancer subtypes. (**a**) *BIRC5* mRNA expression (log_2_-transformed absolute values) in IDH-mutant oligodendroglioma (n = 169), IDH-mutant astrocytoma (n = 258), and IDH-wildtype glioblastoma (n = 229). Glioblastoma (IDH-wildtype) tumors express significantly higher *BIRC5* compared with other subtypes [22]. (**b**) Violin plots depict the distribution of *BIRC5* expression (log_2_-transformed) across the four molecular subtypes of breast cancer: hormone-positive (HR^+^) (n = 515), human epidermal growth factor receptor 2-positive (HER2-positive) (n = 30), triple-positive (TPBC: estrogen receptor positive, progesterone receptor positive, and HER2-positive [23]) (n = 60), and triple-negative (TNBC) (n = 123). TNBC tumors exhibit significantly higher *BIRC5* expression compared with HR^+^, HER2-positive, and TPBC tumors. Adjusted *p*-values are as follows: * *p* < 0.05; ** *p* < 0.01; **** *p* < 0.0001. Data obtained from The Cancer Genome Atlas (TCGA) and accessed through UCSC Xena [24].

Among various cancers, survivin is frequently upregulated in glioblastoma and breast cancer [9,10,11]. Glioblastoma, the most prevalent malignant brain tumor in adults, has a median survival of less than 15 months despite multi-modal therapy [12]. Recurrence is typical, and prognosis is poor, highlighting the need for new or alternative therapeutic approaches. In glioblastoma, both nuclear and cytoplasmic survivin have been detected in primary and recurrent tumors [13]. Notably, survivin expression correlates with glioma grade, with the highest expression observed in glioblastoma, IDH-wildtype, WHO grade 4 [14] (Figure 2a). Even among glioblastomas, with their short overall survival, survivin expression is a negative prognostic factor [15], indicating it may be a viable therapeutic target. Therapeutics developed for brain tumors have additional challenges, including regional differences in the integrity of the blood–brain barrier, tumor heterogeneity, immune cell distribution, immune suppression, and a paucity of immune effectors capable of mediating tumor cytotoxicity (see review article PMID: 34117475 for in-depth discussion). There are a number of emerging pharmacological and surgical strategies that have been developed and are entering late-stage clinical trials that can overcome some of these limitations (PMID: 39862873).

Breast cancer is the leading cause of cancer death in women due to its high rate of metastasis and resistance to existing therapies [16]. Brain metastases occur in approximately 20–40% of breast cancer patients and are associated with poor survival [17]. Surgery and radiotherapy can prolong survival for several years for stage IV breast cancer patients with brain metastases [18], but many patients experience major complications, including a decline in neurological function, and will ultimately succumb to the disease. Temozolomide, known for its effectiveness in penetrating the blood–brain barrier, has been evaluated in phase II trials but was not found to impact overall survival [17], outlining a need for combination therapies. Leptomeningeal disease (LMD), characterized by metastatic cancer involving the subarachnoid space, can also be associated with breast cancer, especially during late stages of the disease. Although LMD is rare, its progression is extremely fast, with a median overall survival of approximately 1 month without treatment [19]. Standard of care consists of radiation therapy and chemotherapy, yet overall survival is only increased by a few months [20]. Metastasis to the central nervous system (both brain parenchymal and LMD) is more common among certain subtypes of breast cancer and occurs in almost 1 out of 3 patients with stage IV human epidermal growth factor receptor-2-positive (HER2-positive) or triple-negative breast cancer [21]. Notably, *BIRC*5 expression varies significantly by breast cancer subtype, with the highest expression in triple-negative cases (Figure 2b). Targeting *BIRC5*/survivin as a therapeutic strategy for cancer treatment has become increasingly attractive, as summarized in Table 1, because of its upregulation in both gliomas and breast cancer and its essential role in inhibiting cell death.

**Table 1:** Clinical landscape of survivin-targeting therapeutics in multiple cancer types.

Trial Identifier *	Drug/Intervention	Mechanism	Phase	Cancer Type(s)	Key Outcomes
NCT04272203 [25]	ABBV-184	Bispecific T-cell engager	1	Acute Myeloid Leukemia, Non-Small Cell Lung Cancer	Well tolerated; evidence of CD3 engagement and cytokine increases.
UMIN000012146 [26]	SVN-2B	Peptide vaccine	2	Pancreatic Adenocarcinoma	No significant change in progression-free survival; evidence of immune responses.
NCT05243524 [27]	Maveropepimut-S	Lipid depot-based vaccine	2b, single arm	Ovarian Cancer	Maveropepimut-S plus cyclophosphamide was well tolerated; durable clinical benefit in recurrent ovarian cancer.
NCT02455557 [28]	SurVaxM	Peptide-mimic vaccine	2a	Newly diagnosed Glioblastoma	SurVaxM plus temozolomide was well tolerated; increase in median overall survival (25.9 months); evidence of survivin-specific immune responses; clinical benefit in patients with methylated and unmethylated tumors.
NCT01333046 [29]	Tumor-Associated Antigen (TAA)-Specific Cytotoxic T Lymphocytes	Adoptive cell therapy	1	Hodgkin/Non-Hodgkin Lymphoma	Targeting all TAA-specific cytotoxic T lymphocytes simultaneously was safe; no dose-limiting toxicities; established recommended phase 2 dose.
NCT01915524 [30]	BI1361849 (CV9202)	mRNA vaccine	1b	Non-Small Cell Lung Cancer	Well tolerated; observed measurable immune responses.
NCT01012102 [31]	EMD640744	Peptide vaccine	1	Solid tumors	Well tolerated; no dose-dependent effects; observed survivin-specific T-cell responses.
NCT02851056 [32]	DC:Ad-S	Dendritic cell vaccine	1	Multiple Myeloma	Well tolerated; no serious adverse events; observed survivin-specific humoral responses.
NCT01038804 [33]	YM155	Small molecule inhibitor	2	HER2-negative metastatic breast cancer	YM155 plus docetaxel was well tolerated; no significant differences compared with docetaxel alone.

*: NCT—National Clinical Trial; UMIN—University Hospital Medical Information Network (Japan).

## 3. Survivin Mediates Resistance to Radiation and Chemotherapy

Radiation has been shown to induce the phosphorylation of survivin, a more stable isoform of the protein, leading to a radioresistant phenotype in glioblastoma cells. When cells are irradiated, they express survivin throughout the cell cycle and not exclusively during the G2/M phases [34]. Another study showed that survivin inhibition augmented radiosensitivity in glioma cells and that the degree of radiosensitization was a function of p53 mutational status secondary to the role of p53 in maintaining chromosomal stability. In the setting of mutated p53, inhibition of survivin triggered extreme centrosome amplification and mitotic cell death rather than apoptosis, indicating that survivin has a role in regulating cytokinesis and chromosome segregation [35].

The propensity of radiation to induce survivin has been exploited to enhance virotherapy against glioma stem cells. For example, we developed a novel oncolytic adenovirus, CRAd-Survivin-pk7, which showed significant toxicity and replication against a panel of passaged and primary CD133^+^ glioma stem cells in vitro. The toxicity associated with CRAd-Survivin-pk7 oncolytic adenovirus was increased from 20% to 50% (*p* < 0.05) after treatment with radiation. In vivo treatment of U373MG CD133^+^ stem cells with CRAd-Survivin-pk7 and radiation significantly inhibited tumor growth (*p* < 0.05), indicating that low-dose radiotherapy can enhance the activity of an oncolytic adenovirus using the radio-inducible survivin promoter [36].

Since survivin is a key mediator of resistance to both radiation and chemotherapy in cancer that is tightly regulated, it is likely that a number of checks and balances are in place to regulate its expression, including regulation by miRNAs. One identified regulatory element is miR-138, a tumor-suppressive miRNA that is downregulated in glioblastoma. Ectopic overexpression of miR-138 was shown to sensitize glioblastoma cells to temozolomide and increase apoptotic cell death. miR-138 was found to directly repress the expression of survivin to enhance caspase-induced apoptosis upon temozolomide treatment. Using an orthotopic xenograft murine model of glioblastoma, the combination of miR-138 with temozolomide was shown to increase survival relative to mice treated with temozolomide alone [37]. However, miR-138 has other regulatory roles, including CTLA-4 and PD-1. In vivo miR-138 treatment of GL261 gliomas in immune-competent mice demonstrated marked tumor regression, a 43% increase in median survival time (*p* = 0.011), and an associated reduction in intratumoral FoxP3^+^ regulatory T cells, CTLA-4, and PD-1 expression. This effect was absent in immune-deficient mice and after CD4^+^ or CD8^+^ T-cell depletion. Moreover, miR-138 showed no suppressive activity on glioma cells at physiological in vivo doses [38], indicating that miR-138 does not exclusively target survivin. Notably, temozolomide is not a potent direct cytotoxic agent [39], and the role of survivin in mediating therapeutic resistance to other agents in glioblastoma, especially those that mediate potent apoptosis such as paclitaxel [40], panobinostat [41], and marizomib [42], is unknown.

## 4. Survivin-Specific Immune Responses

Previous studies have shown survivin to be immunogenic and capable of inducing antitumor immune responses. Although survivin is mainly an intracellular protein, it can be degraded by the proteasome and presented in the context of MHC class I molecules. This presentation effectively recruits and activates CD8^+^ cytotoxic T lymphocytes (CTLs). The clinical relevance of this immunogenicity has been demonstrated by multiple groups. For example, immune responses were observed in cancer patients after receiving a survivin-targeting peptide vaccine conjugate, SurVaxM [28,43]. This vaccine is currently being evaluated in a phase 2 trial for glioblastoma (NCT05163080). Another study identified two survivin-derived peptide epitopes that induced CTL responses in leukemia and melanoma patients [44]. Survivin-specific T cells were also detected in the blood and tumors of melanoma and breast cancer patients, suggesting the ability of survivin-specific T cells to migrate to the effector site and execute antitumor immune responses [45]. Because survivin is immunogenic and essential for apoptosis regulation, it represents a promising target for cancer immunotherapy. Survivin-targeted therapies may be especially powerful in combination with immune checkpoint inhibitors, where priming the immune system could improve sensitivity to checkpoint blockade. However, survivin may also exhibit immunosuppressive activity. In a mouse model of cervical cancer, high expression of survivin correlated with IL-10-producing B cells that have immune-suppressive functions and interfere with antitumor responses [46]. Additionally, extracellular survivin and survivin-containing lymphoma exosomes were found to impair natural killer cell function [47]. Notably, other immune-suppressive mechanisms have not been well studied, and there is limited in vivo data to support generalizability. In the absence of a clearly defined pro-tumor role of cell surface survivin, anti-survivin immunotherapies lack therapeutically relevant mechanisms, and their effects would be no different from those achieved by targeting any other surface antigen under similar conditions.

## 5. What Is the Most Appropriate Type of Therapy to Successfully Target Survivin

Numerous clinical trials of survivin-based therapies have been completed in the past several decades, encompassing a variety of strategies from peptide vaccines to bispecific proteins, with the majority focused on membrane surface targeting (Table 1).

One of the more recent examples is ABBV-184, which is a novel bispecific protein that combines a survivin-specific peptide-targeting T-cell receptor with an anti-CD3 domain. In preclinical studies, T-cell activation and cytotoxicity in HLA-A2:01-positive tumor lines were demonstrated. Clinical evaluation of ABBV-184 as a monotherapy in patients with acute myeloid leukemia and non-small cell lung cancer showed ABBV-184 was well tolerated, with signs of CD3 engagement and transient increases in cytokine levels (NCT04272203) [25]. However, it is unknown if ABBV-184 will be advanced into later-stage clinical trials. A distinct advantage of selecting hematological cancers like leukemia is that therapeutic distribution is likely more uniform relative to the challenges of heterogeneous solid cancer tumor microenvironments. Immune cell distribution and inactivation were likely a confounder for a prior phase II clinical trial investigating a survivin 2B peptide (SVN-2B) in heavily pretreated patients with advanced pancreatic adenocarcinoma (UMIN000012146). A total of 83 patients were enrolled and received either SVN-2B plus IFNβ (n = 30), SVN-2B (n = 34), or placebo (n = 19), but there was no significant improvement in progression-free survival. However, a subgroup analysis indicated that extending the SVN-2B plus IFNβ vaccination strategy could potentially provide a survival benefit [26].

Signals of clinical response were possible in two different survivin vaccine strategies in patients with solid cancers. The first, maveropepimut-S, is a lipid-based peptide vaccine that incorporates survivin-derived epitopes, a universal T helper peptide, and a polynucleotide adjuvant encapsulated in liposomes and formulated in the hydrophobic carrier Montanide ISA51 VG. The clinical trial, DeCidE1, was a multicenter, randomized, open-label, single-arm phase II study that assessed the combination of maveropepimut-S with cyclophosphamide in patients with recurrent ovarian cancer (NCT05243524). A total of 22 patients were enrolled. Within this evaluable cohort, the objective response rate (proportion of patients with complete or partial response) was 21% [90% confidence interval (CI), 7.5–41.9%], while the disease control rate (proportion of patients with complete or partial response or stable disease) was 63% (90% CI, 41.8–81.3%). Notably, 4 individuals with stable disease maintained clinical benefits for up to 25 months [27]. The second, SurVaxM, is a peptide-mimic vaccine evaluated in a cohort of 64 resected newly diagnosed glioblastoma patients (NCT02455557). The combination of SurVaxM with temozolomide was well tolerated with no serious adverse events. Among the 63 patients evaluated, 60 (95.2%) remained progression-free for 6 months after diagnosis. The median progression-free survival was 11.4 months, and the median overall survival was 25.9 months [28]. Notably, SurVaxM induced survivin-specific CD8^+^ T-cell and humoral responses. Clinical activity was evident in patients with MGMT methylated and unmethylated tumors, a response biomarker to temozolomide [48]. A randomized trial of SurVaxM is currently ongoing (NCT05163080). The prior study of SurVaxM in recurrent malignant glioma showed both cellular and humoral immune responses to the vaccine in 6 out of 8 subjects. The vaccine also stimulated HLA-A*02-, HLA-A*03-, and HLA-A*24-restricted T-cell responses. Three patients had either partial clinical response or stable disease for more than 6 months. Median progression-free survival was 4.4 months, and median overall survival was 22 months, with seven of nine patients surviving longer than 12 months [43]. Notably, these trials were conducted with concurrent chemotherapy, which likely contributed to the more favorable outcomes. Therefore, it is unclear what the contribution to survival is with the survivin vaccines. For vaccine and immunotherapy strategies targeting heterogenously expressing EGFRvIII in glioblastoma, treatment failure was secondary to loss of the targeting antigen [49,50,51]. The persistence of survivin expression in these clinical trials was not evaluated, but treatment failure as a function of loss of survivin expression is certainly possible.

To overcome the challenges of tumor antigen heterogeneity, several groups have incorporated targeting survivin alongside other targets. For example, polyclonal T cells reactive to 5 tumor-associated antigens—PRAME, SSX2, MAGEA4, SURVIVIN, and NY-ESO-1—were evaluated in 32 lymphoma patients (NCT01333046). Treatments were well tolerated, with no dose-limiting toxicities observed in either the antigen or dose-escalation phases. Although the maximum tolerated dose was not reached, the highest tested dose associated with clinical activity (two infusions, 2 × 10^7^ cells/m^2^) was selected as the recommended phase II dose. Among the patients with chemo-refractory lymphomas, 2 of 7 with Hodgkin lymphomas and 4 of 8 with non-Hodgkin lymphomas achieved durable complete remission for more than 3 years [29]. Similarly, CV9202, an RNActive^®^-based cancer immunotherapy that encodes survivin and other tumor-associated antigens—New York esophageal squamous cell carcinoma-1, melanoma antigen family C1/C2, trophoblast glycoprotein, and Mucin-1. CV9202 combined with local radiation was evaluated in a phase Ib trial in patients with stage IV non-small cell lung cancer (NCT01915524). Patients received two injections of CV9202, local radiation, and additional injections of CV9202 until disease progression. Treatments were well tolerated, with injection site reactions and flu-like symptoms as the primary adverse events. Antigen-specific immune responses were observed in 84% of patients, and 46.2% achieved stable disease [30]; however, survivin-specific immune response profiling was not conducted in normal subjects or untreated patients. As such, it is unclear if these immune responses are specific to the therapy or the underlying cancer status.

More recently, survivin has been a target, among others, in dendritic cell immune therapeutics and multi-epitope vaccines such as EMD640744. Of 49 solid cancer patients evaluated, 61% of patients showed survivin-specific immune responses, providing evidence for de novo immune induction. However, the best overall response to the vaccine was stable disease (28%) (NCT01012102) [31]. DC:Ad-S, a dendritic cell survivin vaccine, was evaluated in a phase I first-in-human trial in multiple myeloma patients receiving autologous stem cell transplants (NCT02851056). The vaccine was designed to boost humoral and cellular immunity to survivin, thereby potentially eradicating survivin-expressing cells that mediate resistance to apoptosis. The vaccine was given to 14 newly diagnosed patients 7–30 days prior to stem cell collection and 20–34 days post-transplant. Out of the 13 patients included in the analysis, no serious adverse events were identified. Notably, 9 (69%) patients showed an increase in detectable survivin-specific antibodies, and 11 (85%) showed survivin-specific cellular or humoral immune responses. Seven patients showed improved clinical response at day +90, and 6 of these patients remained event-free after 4.2 years. The estimated progression-free survival at 4 years was 71% [32]. When the entire survivin immunotherapy portfolio is assessed, most studies indicate that immunological responses can be generated against survivin, but it is unclear if the induced anti-survivin effector responses are maintained in the tumor microenvironment and if there is sufficient immune effector response distribution in the solid cancers that have been treated.

YM155, a selective cell-permeable survivin inhibitor that does not rely on immunological responses, was evaluated in a phase I clinical study. Patients with EGFR tyrosine kinase inhibitor (TKI)-refractory advanced non-small cell lung cancer received YM155 plus erlotinib (8.0 mg/m^2^/day) every 3 weeks. In preclinical studies, it was observed that downregulation of survivin reversed EGFR TKI resistance and synergistically inhibited tumor growth when combined with erlotinib. Patients treated with this combination exhibited favorable safety and moderate clinical efficacy [52]. In a phase II study, patients with relapsed aggressive B-cell non-Hodgkin lymphoma, who failed or were not candidates for autologous stem cell transplant (n = 34), received continuous infusion of YM155 for 168 h every 3 weeks. The objective response rate was 50%, and the median progression-free survival was 18 months. Median overall survival was not reached at study termination [53]. In a phase II, multicenter, open-label, 2-arm study, patients with stage IV HER2-negative metastatic breast cancer and ≥1 measurable lesion received docetaxel with YM155 or docetaxel alone (NCT01038804) [33]. The median progression-free survival was 8.4 months in the YM155 plus docetaxel group (n = 50) and 10.5 months in the docetaxel alone group (n = 51; HR 1.53; 95% CI 0.83, 2.83; *p* = 0.176). No statistically significant differences were observed for secondary endpoints. However, overall survival was slightly greater in the combo group (630 vs. 601 days; *p* = 0.768). There are no plans to further advance YM155 into later-stage clinical studies. A key limitation across all these studies is that they did not evaluate the association between tumor expression levels of survivin and signals of therapeutic response. Additionally, although breast cancer exhibits the highest level of survivin expression, there has been only one study (the YM155 trial) that included breast cancer patients.

## 6. Next Steps to Optimize Survivin-Targeting Therapeutics

### 6.1. Strategies for Coupling Survivin-Targeting with Immune Therapy

Survivin can induce T-cell-mediated immunity and survivin-specific humoral responses. However, it is unclear if these induced immune responses sufficiently trafficked to the tumor microenvironment, were sufficiently distributed, and/or if the effector responses were maintained in the setting of tumor-mediated immune suppression. As newer survivin-targeting therapeutics are being developed, the contribution of the immune system to antitumor activity can be assessed in immunocompetent and immunocompromised models and/or with in vivo depletion. If the therapeutic effect of the agent is markedly diminished in immunocompromised models, immunotherapeutic combinations could be prioritized for further preclinical vetting. The underlying immune mechanisms can be characterized through ex vivo assays in tumor-bearing mice treated with the anti-survivin agent, assessing survivin-specific antibody induction in serum by ELISA and T-cell reactivity using survivin tetramer staining. If therapeutic activity is not diminished in the immune-deficient background, this implies that direct antitumor activity is not mediated through the immune system, and perhaps other standard treatments like radiation should be prioritized. These types of analysis guide biomarker selection and potential combinatorial strategies. However, it is important to consider that although preclinical cancer models have been used for decades, they may not predict clinical outcomes. These models often fail to capture the biological and molecular heterogeneity of human tumors, and ultimately, clinical trials in patients will be required to determine the therapeutic value of such approaches.

### 6.2. Determine the Role of Radiation Therapy on Survivin Expression for Potential Combination Therapy

It remains unclear whether ionizing radiation downregulates or induces survivin expression, as reported findings are inconsistent and may depend on tumor lineage or radiation dose. Survivin has been proposed as a radiation resistance factor, with reports indicating up to a tenfold increase in its expression following radiation exposure in glioblastoma (GBM) cells [34,54,55]. However, others have shown a contrasting effect, where survivin expression is reduced in certain glioma patient-derived cells, such as the diffuse intrinsic pontine glioma (DIPG) patient-derived cell (PDC) line SF7761, compared to other DIPG PDCs that typically exhibit survivin upregulation after irradiation [56]. Consistent with these discrepancies, our analyses of GBM PDCs revealed heterogeneous survivin responses following radiation exposure. For example, in GBM39 cells, survivin expression was markedly downregulated after radiation, whereas most other GBM PDCs demonstrated significant upregulation at both the mRNA and protein levels (unpublished internal result). Survivin can enhance DNA double-strand break (DSB) repair by either facilitating DSB homologous recombination (HR) [57] or by directly interacting and enhancing DNA-dependent protein kinase catalytic subunit (DNA-PKcs) activities and therefore facilitating nonhomologous end-joining (NHEJ) [58]. It is plausible that distinct tumor cell populations preferentially engage distinct DNA repair pathways in response to radiation, leading to divergent patterns of survivin regulation. Cells with intact DSB repair may require survivin upregulation, whereas others relying on alternative mechanisms of radioresistance may not. Importantly, the formation and composition of DSB repair complexes can be quantified using multiplex immunofluorescence in irradiated tissues, allowing investigation of potential correlations between survivin expression and DSB repair activity. Such analyses may also provide mechanistic insights to guide the optimal scheduling of survivin-targeting therapies in combination with radiation.

### 6.3. Determine if Threshold Levels of Survivin Expression Are Required for Therapeutic Response

It is unknown why survivin expression is highest in two markedly different cancer lineages—tumors arising from distinct cellular and developmental origins, such as breast cancer and glioblastoma. Modulation of survivin expression across various cancer lineages could further probe the more specific roles this pathway plays, or not, in mediating tumorigenic versus immunogenic roles. Previous clinical trials did not assess how survivin expression correlates with therapeutic response. To address this, survivin expression could be eliminated from cancer cell lines using CRISPR. Parental and survivin knockout cancer cell lines would then be implanted at varying ratios to first verify that survivin expression does not alter in vivo growth kinetics. If survival curves are similar, each cohort would then be randomized to be treated with the anti-survivin therapeutic. If there is a clear threshold of expression associated with the response to the therapeutic, this will inform the optimal cut-point for companion biomarker design. Future studies should also be directed to determining whether the elimination of survivin induces alternative anti-apoptotic compensatory mechanisms that support equivalent growth kinetics.

### 6.4. Ascertain If Survivin Expression Is Differentially Expressed in Human Primary Versus Brain Metastasis

Although membrane-associated signaling of survivin may be tumorigenic and drive metastasis, the underlying mechanisms need to be elucidated in future studies. Matched human primary and metastatic tumor samples from cancer patients would help identify the disease stage at which patients are most likely to benefit from a survivin-targeted therapeutic. Given the known high expression of survivin in primary breast cancers, this lineage may be the most appropriate focus and should include samples spanning multiple molecular subtypes as well as breast cancer brain metastases. Quantifying the number of survivin-expressing cells will clarify whether expression levels are consistent across organ sites and subtypes. In addition, clinical treatment variables such as prior chemotherapy and/or radiation may be associated with survivin expression levels and should be considered in the analyses.

## 7. Conclusions

Despite decades of study and development, survivin-targeted therapies have yet to achieve durable clinical success. There are several limitations that have constrained prior clinical studies, including (1) the lack of biomarker-driven patient stratification that did not link survivin expression to therapeutic response; (2) limited understanding of how survivin may modulate and prime the immune system and if these responses participate in clinical benefit; and (3) insufficient characterization of how survivin expression is regulated following radiation, despite evidence of its contribution to radioresistance. Addressing these gaps and pitfalls will be critical to refining the future clinical potential of targeting survivin. Future studies should incorporate survivin expression into clinical design, ascertain if there is a threshold for survivin expression and therapeutic response, and evaluate survivin expression longitudinally and in response to radiation and other treatment strategies. Advancing these efforts may be valuable in transforming survivin-targeted therapies into clinically meaningful interventions across gliomas, breast cancer and other malignancies.

## Data Availability

No new data were created or analyzed in this study. Data sharing is not applicable to this article.
